# Population pharmacokinetics in phase I drug development: a phase I study of PK1 in patients with solid tumours

**DOI:** 10.1038/sj.bjc.6690657

**Published:** 1999-09

**Authors:** A H Thomson, P A Vasey, L S Murray, J Cassidy, D Fraier, E Frigerio, C Twelves

**Affiliations:** Department of Medicine & Therapeutics, University of Glasgow, CRC Department of Medical Oncology Beatson Oncology Centre, Western Infirmary, Glasgow, G11 6NT, UK; Bioanalytical Laboratory Pharmacokinetics and Metabolism Department, Pharmacia & Upjohn, Nerviano, Italy; Department of Medicine & Therapeutics, Institute of Medical Sciences, Foresterhill, Aberdeen, AB25 2ZD, UK

**Keywords:** PK1-doxorubicin, solid tumours, population pharmacokinetics

## Abstract

Doxorubicin pharmacokinetics were determined in 33 patients with solid tumours who received intravenous doses of 20–320 mg m^−2^ HPMA copolymer bound doxorubicin (PK1) in a phase I study. Since assay constraints limited the data at lower doses, conventional analysis was not feasible and a ‘population approach’ was used. Bound concentrations were best described by a biexponential model and further analyses revealed a small influence of dose or weight on V1 but no identifiable effects of age, body surface area, renal or hepatic function. The final model was: clearance (Q) 0.194 l h^−1^; central compartment volume (V1) 4.48 × (1+0.00074 × dose (mg)) l; peripheral compartment volume (V2) 7.94 l; intercompartmental clearance 0.685 l h^−1^. Distribution and elimination half-lives had median estimates of 2.7 h and 49 h respectively. Free doxorubicin was present at most sampling times with concentrations around 1000 times lower than bound doxorubicin values. Data were best described using a biexponential model and the following parameters were estimated: apparent clearance 180 l h^−1^; apparent V1 (l) 1450 × (1+0.0013 × dose (mg)), apparent V2 (l) 21 300 × (1–0.0013 × dose (mg)) × (1+2.95 × height (m)) and apparent Q 6950 l h^−1^. Distribution and elimination half-lives were 0.13 h and 85 h respectively. © 1999 Cancer Research Campaign


*British Journal of Cancer* (1999) **81**(1), 99–107
© 1999 Cancer Research Campaign
Article no. bjoc.1999.0657

### Population pharmacokinetics in phase I drug development: a phase I study of PK1 in patients with solid tumours

**AH Thomson[1], PA Vasey[2], LS Murray[1], J Cassidy[4], D Fraier[3], E Frigerio[3] and C Twelves[2]**

[1]Department of Medicine & Therapeutics, University of Glasgow, [2]CRC Department of Medical Oncology, Beatson Oncology Centre, University of Glasgow, Western Infirmary, Glasgow G11 6NT, UK; [3]Bioanalytical Laboratory, Pharmacokinetics and Metabolism Department, Pharmacia & Upjohn, Nerviano, Italy; [4]Department of Medicine & Therapeutics, Institute of Medical Sciences, University of Aberdeen, Foresterhill, Aberdeen AB25 2ZD, UK

**Summary** Doxorubicin pharmacokinetics were determined in 33 patients with solid tumours who received intravenous doses of 20–320 mg $m^{-2}$ HPMA copolymer bound doxorubicin (PK1) in a phase I study. Since assay constraints limited the data at lower doses, conventional analysis was not feasible and a ‘population approach’ was used. Bound concentrations were best described by a biexponential model and further analyses revealed a small influence of dose or weight on V1 but no identifiable effects of age, body surface area, renal or hepatic function. The final model was: clearance (Q) 0.194 l $h^{-1}$; central compartment volume (V1) 4.48 × (1+0.00074 × dose (mg)) l; peripheral compartment volume (V2) 7.94 l; intercompartmental clearance 0.685 l $h^{-1}$. Distribution and elimination half-lives had median estimates of 2.7 h and 49 h respectively. Free doxorubicin was present at most sampling times with concentrations around 1000 times lower than bound doxorubicin values. Data were best described using a biexponential model and the following parameters were estimated: apparent clearance 180 l $h^{-1}$; apparent V1 (l) 1450 × (1+0.0013 × dose (mg)), apparent V2 (l) 21 300 × (1–0.0013 × dose (mg)) × (1+2.95 × height (m)) and apparent Q 6950 l $h^{-1}$. Distribution and elimination half-lives were 0.13 h and 85 h respectively.

**Keywords:** PK1-doxorubicin; solid tumours; population pharmacokinetics

Prague-Keele 1 (PK1) is a novel cytotoxic drug in which doxorubicin is complexed with *N*-(2-hydroxypropyl) methacrylamide (HPMA), a water-soluble polymer, by a peptidyl linker. The rationale behind this compound is that whereas the vasculature of the normal circulation prevents high molecular weight macromolecules from crossing cells by passive diffusion, tumour vessels are ‘leaky’, which allows the compound to enter the tumour (Matsumura and Maeda, 1986). Once inside, they are poorly cleared due to an inefficient lymphatic drainage system (Matsumura and Maeda, 1986; Maeda and Matsumura, 1989). PK1 is reported to be stable in plasma, thus potentially protecting normal cells, but when taken up into tumours it is cleaved intracellularly by lysosomal cysteine proteinases to release free doxorubicin (Duncan et al, 1982). This modification can allow much higher doses of doxorubicin to be administered while reducing the potential for toxicity.

In this study, the pharmacokinetics of bound and free doxorubicin were determined from plasma concentrations measured during a phase I dose-ranging trial. Due to sampling and assay limitations, few concentration data were available in patients who received lower doses of this drug and it was therefore not possible to analyse these sparse data by conventional, individual non-linear regression. The aim was therefore to utilize a ‘population approach’, in which all data could contribute to the estimation of pharmacokinetic parameters, to analyse the data. The population approach aims to characterize factors that influence drug handling in individual patients and to estimate both variability in pharmacokinetics between individuals and residual error on concentration measurements. When it was first introduced in the late 1970s it was applied to sparse digoxin data collected from many patients during routine therapeutic drug monitoring (Sheiner et al, 1977). Since then, a number of computer methodologies have been developed and the approach has been applied to data collected using both sparse and extensive sampling protocols in a wide range of therapeutic areas (Yuh et al, 1994). More recently, the population approach has gained support from both the regulatory authorities and the pharmaceutical industry as a useful tool in drug development (Samara and Granneman, 1997; Tett et al, 1998).

*Received 19 November 1998*
*Revised 25 March 1999*
*Accepted 12 April 1999*

*Correspondence to:* AH Thomson

### MATERIALS AND METHODS

### Protocol

Data were available from a phase I dose-ranging study of PK1 in 36 patients with histologically confirmed solid tumours considered refractory or resistant to conventional treatments. The study was approved by the Hospital Research and Ethics Committee and each patient gave informed, written consent before they participated. All patients had a performance status of 2 or better on the Eastern Co-operative Oncology Group–Zubrod–WHO scale and had adequate bone marrow, hepatic and renal function as evidenced by: neutrophils ≥ 2000 $mm^{-3}$; platelets ≥ 100 000 $mm^{-3}$; haemoglobin ≥ 10 g $l^{-1}$; bilirubin < 20 μmol $l^{-1}$; aspartate transaminase/alanine transaminase < 2 × the upper limit of normal; creatinine < 150 μmol $l^{-1}$. Full details of the study and clinical outcomes have been presented elsewhere (Vasey et al, 1999).

PK1 was supplied as a freeze-dried lyophilized powder in glass vials containing 50 mg of doxorubicin-equivalent bound to approximately 530 mg of polymer. This was reconstituted before use with 25 ml 0.9% saline for injection to give a final concentration of 2 mg $ml^{-1}$. Doses started at 20 mg $m^{-2}$ and were escalated according to clinical criteria using a modified Fibonacci scheme. Three patients each received 20, 40, 80 and 120 mg $m^{-2}$ and 6 each received 180, 240, 280 and 320 mg $m^{-1}$. Doses were administered every 3 weeks until withdrawal from the study. PK1 was given as a slow bolus over 5 min at low doses (20, 40 and 80 mg $m^{-2}$), as an infusion over 0.5–0.75 h at intermediate doses (120 and 180 mg $m^{-2}$) and over 1–1.5 h at high doses (240, 280 and 320 mg $m^{-2}$). Samples were withdrawn before the first dose, at 0.25 h during the infusion (doses > 80 mg $m^{-2}$), at the end of the infusion, then at the following times after the end of the infusion: 0.08, 0.16, 0.25, 0.33, 1, 2, 4, 8, 12 and 24 h. Further samples were withdrawn at 48, 72, 96, 120, 168, 192 and 360 h in a number of patients.

The following data were stored in a spreadsheet file for analysis: patient identification number; dose (mg); infusion rate (mg $h^{-1}$); time from the start of the infusion; bound doxorubicin concentration; performance status; age; height; weight; body surface area; sex; concentrations of bilirubin, albumin, aspartate aminotransferase, alanine aminotransferase and creatinine; and estimated creatinine clearance (Cockcroft and Gault, 1976). A similar file was then created containing the free doxorubicin concentration measurements.

### Assay

Bound and free doxorubicin concentrations were measured by high-performance liquid chromatography (HPLC) with fluorescence detection by the Bioanalytical Laboratory, Pharmacokinetics and Metabolism Department, P & U, Nerviano, Italy. Details of the assay method have been described in detail elsewhere (Fraier et al, 1995; Vasey et al, 1999). Limits of quantification for free and bound doxorubicin were 0.38 ng $ml^{-1}$ and 5.1 ng $ml^{-1}$ respectively, and interday assay coefficient of variation ranged from 7.8 to 10.8% for free and 6.3 to 10.7% for bound doxorubicin.

### Data analysis

The pharmacokinetics of bound and free doxorubicin were determined separately using a population approach in which concentrations from all patients were analysed simultaneously. Models were fitted to the data using the first order estimation method in the package NONMEN (Version IV) (Beal et al, 1994) and FORTRAN compilation was performed using FORTRAN PowerStation Version 1.0a. Precision was set at three significant figures. Drug input was assumed to be zero order (constant rate infusion) and both monoexponential and biexponential elimination models were compared. NONMEM estimated the pharmacokinetic parameters of the structural model, proportionality constants relating parameters (clearance, volume of distribution, etc.) to clinical factors, and random effects, that is interpatient and residual variabilities.

Interpatient variability in the parameter estimates was assumed to correspond to a log-linear model, i.e.

$$P_{ik} = PopP_k \times \exp(\eta_{ik})$$

Where $P_{ik}$ represents the kth pharmacokinetic parameter estimate for the ith individual, $PopP_k$ are the population estimates of the parameters (clearance, volume, etc.) and $\eta_{ik}$ are the individual (i) deviations from the population parameters (k). Covariance between parameters was investigated during model development.

Three models were investigated for residual error:

| | |
|---|---|
| Additive | $c_{ij} = pred_{ij} + \varepsilon_{ij}$ |
| Exponential | $c_{ij} = pred_{ij} \times \exp(\varepsilon_{ij})$ |
| Combined | $c_{ij} = pred_{ij} \times \exp(\varepsilon_{1ij}) + \varepsilon_{2ij}$ |

where $c_{ij}$ is the jth measured concentration in the ith individual and $\varepsilon_{ij}$ represent the differences between each measured and predicted ($pred_{ij}$) concentration. These residual errors represent factors such as intrapatient variability, assay error and sampling error.

Individual estimates of the pharmacokinetic parameters were obtained for each patient following the population analysis using the 'posthoc' option in NONMEM. Half-lives were derived from these parameters using standard equations.

Scatterplots of the pharmacokinetic parameters against the available clinical data were examined for obvious trends, and an all subsets multiple linear regression analysis of clinical factors against the pharmacokinetic parameters was undertaken using the statistical package MINITAB. Factors identified by these preliminary approaches as possibly influencing the pharmacokinetics of doxorubicin were then sequentially included in the population model until the best model was identified. The following structure was typically used:

$$PopP_k = \theta_1 \times (1 + \theta_2 \times (factor_1 - median)) \times (1 + \theta_3 \times (factor_2 - median)) \times \ldots \times$$

where $PopP_k$ is the pharmacokinetic parameter (clearance, volume, etc.), $factor_1$, $factor_2$, etc. represents the value of the clinical factor under investigation and θ are the parameters to be estimated.

A number of criteria were taken into account when comparing models. Hierarchical models were compared statistically using a likelihood ratio test. NONMEM produces an objective function value (minus twice the log likelihood of the data) for each model. The difference in these values between two models approximates to a $\chi^2$ distribution with degrees of freedom equal to the difference in the number of model parameters (Beal et al, 1994). Significance was set at $P < 0.005$ (a reduction of > 7.9 for 1 degree of freedom). Differences between the measured and population predicted concentrations (residuals) and also weighted residuals (residuals standardized according to their standard deviation) were plotted against time and predicted concentration, and examined for outliers and trends that might imply model misspecification. Profiles of measured, population predicted and individual predicted concentrations against time for each patient, standard errors of parameter estimates and changes in estimates of intersubject and residual variability were also assessed.

### RESULTS

### Patients

Concentration data were available from 33 patients (18 male) whose ages ranged from 34 to 78 years (median 58 years) and body surface areas from 1.4 to 2.2 $m^2$ (median 1.7 $m^2$). Seven patients had a performance score of 0, 22 had a score of 1 and four had a score of 2. Estimated creatinine clearance ranged from 31 to

© 1999 Cancer Research Campaign

**Table 1** Population pharmacokinetic parameter estimates arising from models fitted to bound doxorubicin concentration data

| | Basic model | Model including weight | Model including dose |
|---|---|---|---|
| Objective function value | 1786.4 | 1774.8 | 1770.9 |
| CL (l $h^{-1}$) | 0.194 | 0.192 | 0.194 |
| % cv | 8.0 | 8.2 | 8.2 |
| Interpatient variability in CL | 35% | 34% | 35% |
| V1 (1) | 4.36 | 4.37 | 4.48 |
| % cv | 6.1 | 5.6 | 4.9 |
| Influence of weight or dose on V1 | | 0.0083 | 0.000737 |
| % cv | | 43 | 27 |
| Interpatient variability in V1 | 34% | 31% | 27% |
| V2 (1) | 7.50 | 7.68 | 7.94 |
| % cv | 8.1 | 8.3 | 9.0 |
| Interpatient variability in V2 | 45% | 46% | 43% |
| Q (l $h^{-1}$) | 0.696 | 0.681 | 0.685 |
| % cv | 11 | 12 | 11 |
| Interpatient variability in Q | 54% | 51% | 56% |
| Residual error 1 | 0.0150 | 0.0146 | 0.0162 |
| Residual error 2 | 1.22 | 1.22 | 1.19 |

*% cv* = coefficient of variation of parameter estimate, CL = clearance, V1 = volume of the central compartment, V2 = volume of the peripheral compartment, Q = intercompartmental clearance. N.B. Interpatient variability is expressed as a percentage coefficient of variation. The residual error model had the form $c_{ij} = pred_{ij} \times \exp(error_{1ij}) + error_{2ij}$ and the covariate model took the form V1 = θ1 x (1 + θ2 x factor) where θ represents the parameter to be estimated and factor represents (dose – median dose) or (weight – median weight).

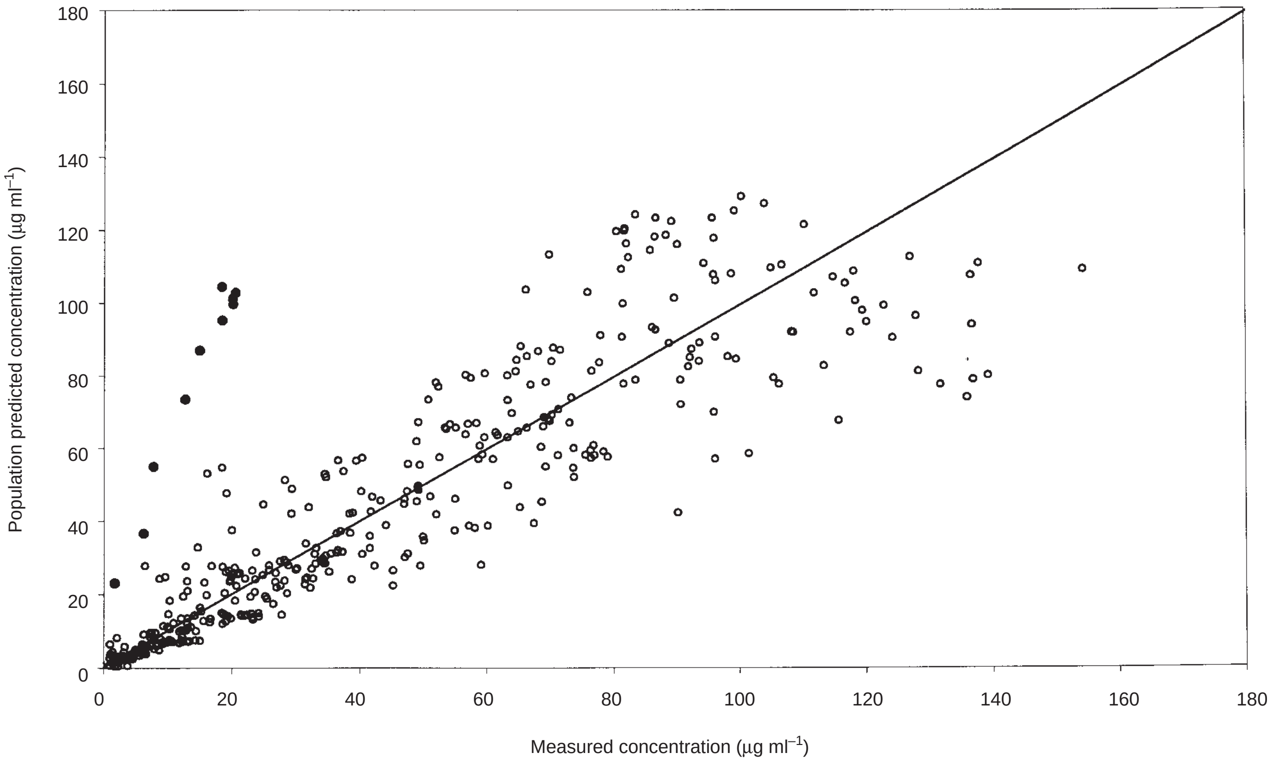

**Figure 1** Population predicted versus measured bound doxorubicin concentrations arising from the basic model. The data from Patient 26 are represented by solid circles

165 ml $min^{-1}$ but only three patients had estimated creatinine clearances below 50 ml $min^{-1}$ (31, 44 and 44 ml $min^{-1}$). Liver biochemistry tests were normal or only minimally disturbed. No patient had elevated bilirubin concentrations, albumin concentrations ranged from 31 to 47 g $l^{-1}$ and the highest concentrations of alanine aminotransferase and aspartate aminotransferase were 57 u $l^{-1}$ and 62 u $l^{-1}$ respectively.

### Bound doxorubicin analysis

Three hundred and eighty-eight bound doxorubicin concentrations were available for analysis. The number of concentrations per patient ranged from 8 to 15 (median 11) and the last sample time ranged from 8 to 385 h post dose. Concentration measurements were available up to 24 h post dose in 17 patients and 29 concentrations were measured more than 90 h post dose.

*© 1999 Cancer Research Campaign* 

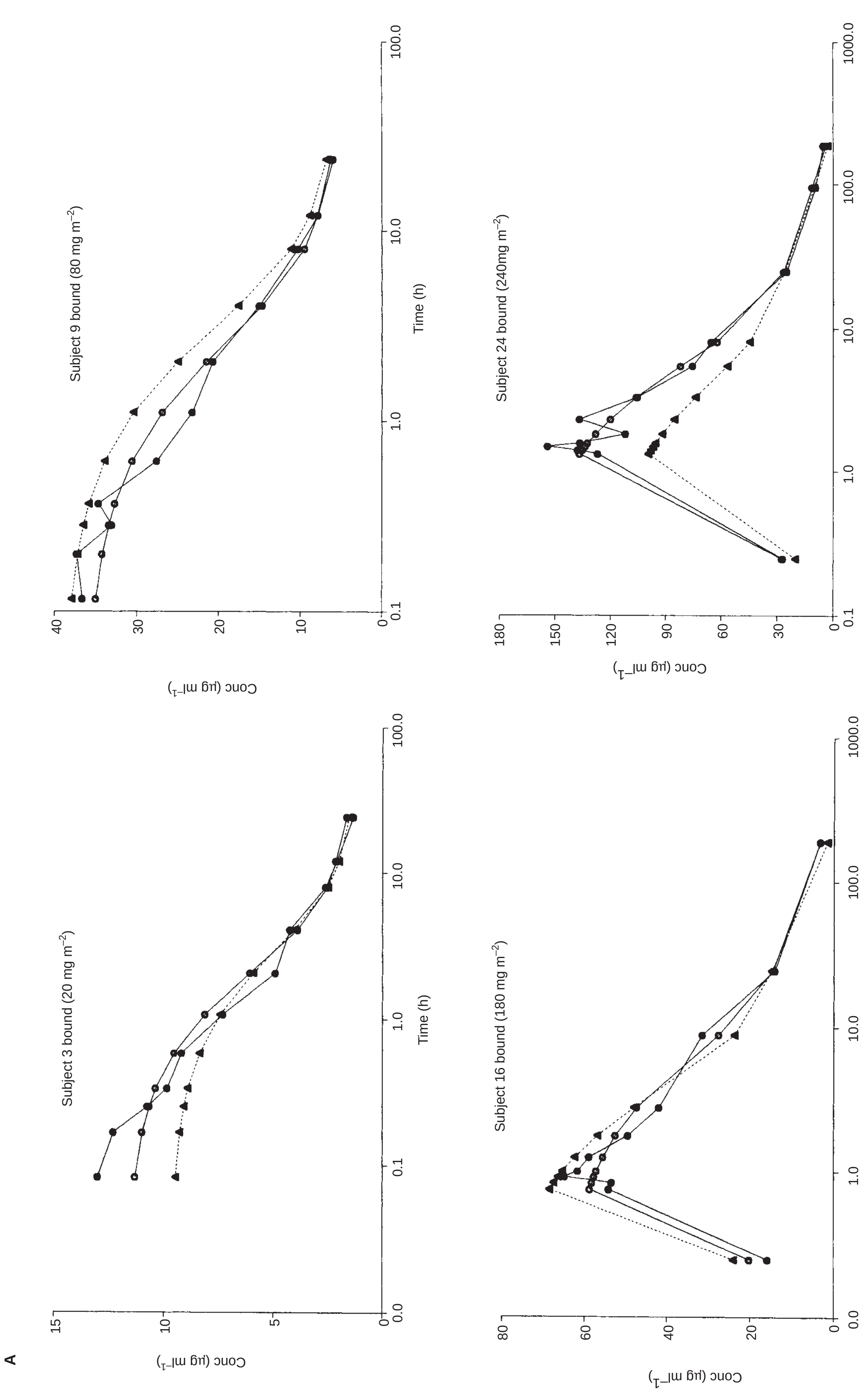

*© 1999 Cancer Research Campaign*

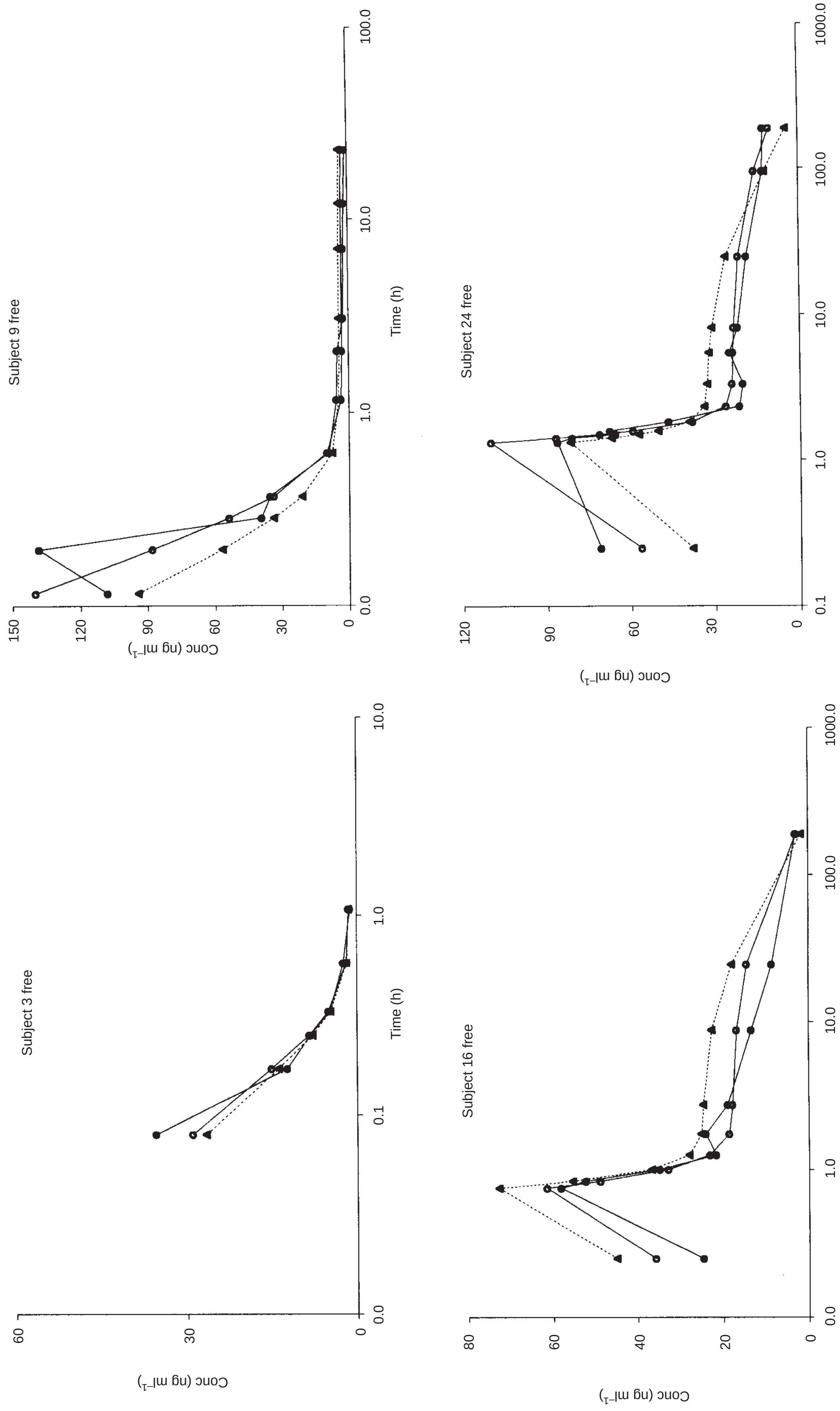

**Figure 2** Bound (**A**) and free (**B**) doxorubicin concentration-time profiles from four patients showing measured (●), population predicted (▲) and individual predicted (○) concentrations from the full models for bound doxorubicin (including dose) and free doxorubicin (model 13). N.B. Time is plotted on a log scale for clarity

**Table 2** Summary of the covariate models tested for free doxorubicin

| Model | Covariate | OBJ | Compared with model | DIFF | Significance |
|---|---|---|---|---|---|
| 1 | None | 2269.5 | | 0.0 | |
| 2 | Dose on V1 | 2252.1 | 1 | 17.4 | $P < 0.005$ |
| 3 | Weight on V1 | 2263.6 | 1 | 5.9 | $P < 0.05$ |
| 4 | BSA on V1 | 2264.9 | 1 | 4.6 | $P < 0.05$ |
| 5 | Height on V1 | 2265.1 | 1 | 4.4 | $P < 0.05$ |
| 6 | Dose on V2 | 2259.3 | 1 | 10.2 | $P < 0.005$ |
| 7 | Weight on V2 | 2265.3 | 1 | 4.3 | $P < 0.05$ |
| 8 | BSA on V2 | 2262.4 | 1 | 7.1 | $P < 0.01$ |
| 9 | Height on V2 | 2244.4 | 1 | 25.1 | $P < 0.005$ |
| 10 | Albumin on CL | 2269.5 | 1 | 0.0 | NS |
| 11 | Dose on V1, height on V2 | 2227.4 | 9 | 17.0 | $P < 0.005$ |
| 12 | Dose on V2, height on V2 | 2217.8 | 9 | 26.6 | $P < 0.005$ |
| 13 | Dose on V1, height on V2, dose on V2 | 2204.0 | 12 | 13.8 | $P < 0.005$ |

OBJ = objective function value, DIFF = difference in objective function values (> 7.9 is significant at $P < 0.005$), V1 = volume of the central compartment, V2 = volume of the peripheral compartment, CL = clearance, BSA = body surface area.

A biexponential elimination model was superior to a mono-exponential model in both objective function value (difference of 229) and weighted residual plots. Allowing covariance between the parameters further improved the fit and residual error was best described by the combined model.

Weighted residuals > 3 are often regarded as outliers (Beal et al, 1994) and one concentration with a weighted residual of 15 was removed from the data set and all subsequent analyses. This was a measurement of 81 µg ml$^{-1}$ after 0.25 h of a 1 h infusion. Since the concentration at the end of the infusion was 86 µg ml$^{-1}$, the initial result (which was 3–4 times higher than concentrations measured at 0.25 h in other patients on the same dose) was highly unlikely.

The population parameter estimates arising from the basic pharmacokinetic model are presented in Table 1. In most patients these population estimates provided a good fit of the individual data, however, the bound concentrations measured in patient 26 were substantially lower than those predicted by the population model. This suggested that this patient was an outlier (Figure 1). In all cases the individual parameter estimates fitted the measured concentrations well, although in five patients small fluctuations in bound doxorubicin concentration were observed in the first 10–20 min after the end of the infusion.

Individual estimates of clearance (CL), volume of the central compartment (V1), volume of the peripheral compartment (V2) and intercompartmental clearance (Q) were plotted against each patient's identification number (ID), dose and clinical characteristics. No obvious trends were seen and there was no evidence of dose-dependency in clearance. Multiple linear regression analysis identified creatinine concentration and creatinine clearance as possibly influencing clearance, and weight and dose as influencing volume, but in all cases the explanatory power of these variables was very low (only 11–15% of the variability in CL or V1).

In the NONMEM analysis indicators of neither renal function nor sex significantly influenced CL, V1 or V2 when included in the population model, however, both dose and weight had statistically significant effects on V1. This influence of dose on V1 persisted even if patient 26 was removed from the data set. A small improvement in fit was obtained when both factors were included in the model but this did not achieve the predetermined level of significance. Population estimates arising from the basic model and the models including weight and dose are shown in Table 1. The model including dose found that V1 changed by 0.07% for every 1 mg above and below 345 mg (the median dose) and produced population estimates of V1 that ranged from 3.4 l at 33 mg (the lowest dose) to 5.6 l at 670 mg (the highest dose). In the model including weight, V1 changed by 0.8% for every kg above and below 67 kg which resulted in an estimated range of 3.4–5.7 l across the weight range of 41–103 kg. Intersubject coefficient in variation in V1 fell from 34% to 31% when weight was included and to 27% when dose was included.

**Table 3** Population pharmacokinetic parameter estimates of free doxorubicin

| | Basic model (model 1, Table 2) | Full model (model 13, Table 2) |
|---|---|---|
| Objective function value | 2269.5 | 2204.0 |
| CL (1 h$^{-1}$) | 179 | 180 |
| % cv | 7.8% | 10% |
| Interpatient variability on CL | 35% | 33% |
| V1 (1) | 1340 | 1450 |
| % cv | 14% | 13% |
| Influence of dose on V1 | | 0.0013 |
| % cv | | 29% |
| Interpatient variability on V1 | 99% | 58% |
| V2 (1) | 16900 | 21 300 |
| % cv | 11% | 8.7% |
| Influence of dose on V2 | | –0.0013 |
| % cv | | 40% |
| Influence of height on V2 | | 2.95 |
| % cv | | 17% |
| Interpatient variability on V2 | 74% | 57% |
| Q (l h$^{-1}$) | 8310 | 6950 |
| | 17% | 12% |
| Interpatient variability on Q | 159% | 128% |
| Residual error | 43% | 40% |

*% cv* = coefficient of variation of parameter estimate, CL = clearance, V1 = volume of the central compartment, V2 = volume of the peripheral compartment, Q = intercompartmental clearance. N.B. Interpatient variability is expressed as a percentage coefficient of variation. The residual error model had the form $c_{ij} = pred_{ij} \times \exp(error_{ij})$ and the covariate models took the form $P = \theta 1 \times (1 + \theta 2 \times factor)$ where P is V1 or V2, θ represents the parameter to be estimated and factor represents (dose – median dose) or (height – median height).

*© 1999 Cancer Research Campaign*

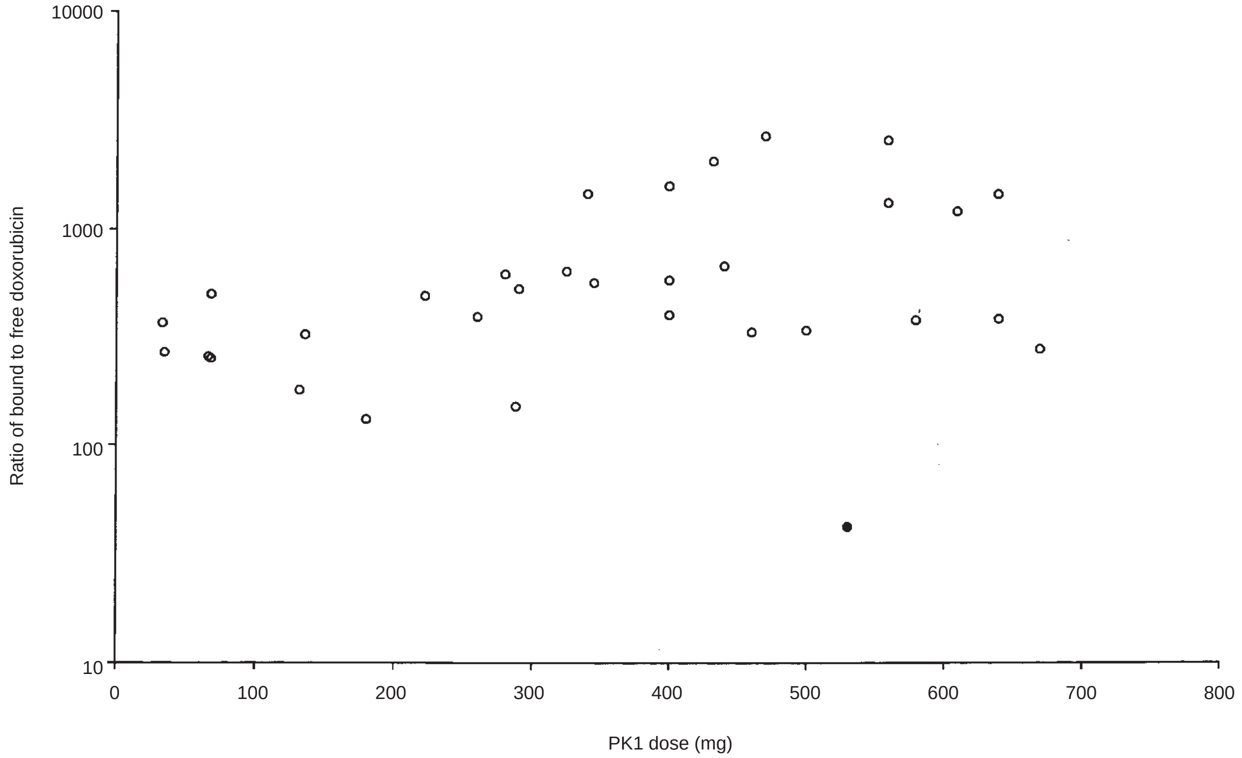

**Figure 3** Ratio of bound to free doxorubicin at the time of the first sample. (Patient 26 is represented by a solid circle.) N.B. The first sample was typically drawn at 5 min for doses < 200 mg and 15 minutes for doses > 200 mg

The population estimate of clearance using the dose model was 0.194 l $h^{-1}$ (Table 1) with individual estimates ranging from 0.147 to 0.647 l $h^{-1}$. Derived Vss (V1 + V2) had a median estimate of 12 l (range 7.3–52 l). Patient 26 had the highest estimates of all the individual parameters. Derived distribution half-life had a median value of 2.7 h (range 1.2–5.1 h) and elimination half-life had a median value of 49 h (range 32–81 h). Figure 2A illustrates the concentration-time profiles for four typical subjects together with the predicted concentrations arising from the population and the 'posthoc' individual parameter estimates.

### Population analysis of free doxorubicin concentrations

Data were available from 33 patients, and comprised 379 concentrations with a median of 11 samples per patient (range 6–15). The time of the last detectable concentration ranged from 1 to 385 h post dose and in five patients was 12 h post dose or less. Twenty-nine samples were above the limit of quantification beyond 90 h post dose. One outlying point, an end of infusion concentration of 515.85 ng $ml^{-1}$, was removed from the analysis. This result was inconsistent with subsequent measurements of 183 ng $ml^{-1}$ (or less) for samples beyond 5 min after the end of the infusion.

Free doxorubicin data were also best described by a biexponential elimination model. An exponential model proved adequate to describe the residual error and it was not possible to estimate covariance between parameters. Population parameter estimates obtained from the basic pharmacokinetic model indicated that free doxorubicin 'clearance' at 179 l $h^{-1}$ was 1000 times the clearance of bound doxorubicin. This apparent clearance probably reflected the small amount of free doxorubicin available when the injection was administered. Although this model provided a satisfactory fit to most data, examination of the individual plots suggested an underestimation of some initial (peak) concentrations at doses up to 120 mg $m^{-2}$. However, good predictions of peak concentrations were achieved at higher doses.

No clear trends were identified when individual estimates of apparent CL, V1, V2 and Q were plotted against clinical characteristics and there was no evidence of dose dependency in CL. Multiple regression analysis suggested that albumin might influence apparent CL, dose and bilirubin might influence apparent V1 and dose and height might influence apparent V2. These factors, and body surface area, were therefore investigated using NONMEM and Table 2 summarizes the results. Statistically significant improvements in fit ($P < 0.005$) were observed with dose on V1 and both dose and height on V2. Smaller effects ($P < 0.05$) were observed with weight, height and body surface area on V1 and weight and body surface area on V2. When factors were combined neither weight nor body surface area improved the fits obtained using dose on V1 and V2 but the combination of dose and height on V2 produced a statistically significant effect. Table 3 shows the parameter estimates from the basic model and the full model with dose on V1 and both dose and height on V2. These estimates indicated a 0.13% change in both V1 and V2 with a 1 mg change in dose from 345 mg and a 29.5% change in V2 with a 0.1 m change in height from 1.7 m. Figure 2B illustrates the measured, population predicted and individual predicted free doxorubicin concentration–time profiles obtained for patients 3, 9, 16 and 24 using this model.

*© 1999 Cancer Research Campaign*

Individual estimates of apparent CL ranged from 107 to 282 l $h^{-1}$ but, due to the paucity of concentration data beyond the distribution phase, individual estimates for patients on low doses (< 80 mg) were essentially unchanged from the population values. Derived apparent Vss had a median estimate of 22 883 l (individual range 2978–43 189 l), the median derived distribution half-life was 0.13 h (range 0.08–1.4 h) and derived elimination half-life had a median of 85 h (range 10–168 h). Patient 26 was again an outlier but in this case had concentrations of free doxorubicin that were consistently higher than predicted by the population model and the lowest estimates of apparent V1 and V2. Although free doxorubicin was generally detectable at the first time point in all patients, the ratio of bound to free doxorubicin in patient 26 was much lower than in other subjects (as illustrated in Figure 3).

### DISCUSSION

A population approach has been used to analyse bound and free doxorubicin data generated during a phase I clinical study of PK1 in patients with solid tumours. Although it has been suggested that this methodology may be useful for dose escalation designs where many concentrations fall below assay limits (Schoemaker and Cohen, 1996), many population studies to date have focused on sparse data collected during Phase III of drug development or routine clinical practice (Samara and Granneman, 1997). The population approach has been applied in cancer chemotherapy, especially for drugs such as carboplatin (Chatelut et al, 1995), docetaxel (Bruno et al, 1996) and etoposide (Nguyen et al, 1998) and in the analysis of phase I data (Chabot et al, 1995; Launay Iliadis et al, 1995). Limited sampling strategies employing Bayesian approaches have also been used to estimate population parameters during early drug development (Jodrell et al, 1994; Reyno et al, 1995; McLeod et al, 1996; Piscitelli et al, 1997). The population approach has particular relevance to phase I drug development in cancer chemotherapy due to the data handling problems that are inherent when very low initial doses (1/10 of the $LD_{10}$) are used. Indeed, attempts were made to analyse the low dose data from the present study using conventional non-linear regression but this proved unsuccessful due to lack of information about the elimination phase. Because the population approach analyses all the data simultaneously, such sparse or incomplete data were not simply discarded but contributed to the estimation of pharmacokinetic parameters. In addition, this initial analysis provides a basis for future studies in which the population model can be developed and refined as more data become available.

Bound doxorubicin concentration measurements were best described by a biexponential disposition model and in most cases the population mean parameter estimates provided good predictions of the measured concentrations. There was no evidence of double peaks or redistribution as has been observed with pegylated-liposomal doxorubicin (Amantea et al, 1997). Clearance was well defined and the inter-individual variability was low at 26%. In contrast, interpatient coefficients of variation in intercompartmental clearance and volume of the peripheral compartment were higher at 54% and 45% respectively.

Scatterplots and multiple linear regression analysis failed to identify clear relationships between clinical characteristics and pharmacokinetic parameters. Doxorubicin is principally cleared by hepatic and biliary routes and reduced clearance has previously been demonstrated in patients with abnormal liver biochemistry tests (Piscitelli et al, 1993). However, the lack of significant clinical factors was not unexpected because the number of patients was relatively small and all had essentially normal renal and hepatic function. Both dose and weight influenced V1 when included as single covariates but combining these factors offered no significant advantage. In contrast to the results of Amantea et al (1997) in a larger group of 43 patients who received pegylated-liposomal doxorubicin, body surface area was not found to influence the clearance of PK1, nor was clearance reduced in female patients, as previously shown by Dobbs et al (1995) with standard doxorubicin. However, a larger number of subjects would be required to investigate these influences properly.

Due to the relative paucity of data, free doxorubicin concentrations were more difficult to interpret than bound concentrations. Although a biexponential model appeared to describe the data well overall, there was an underestimation of peak concentrations in some patients at low doses which may suggest a model misspecification problem or a formulation effect. Previous studies have identified triexponential elimination after the administration of doxorubicin (Bronchud et al, 1990; Piscitelli et al, 1993; Dobbs et al, 1995; Jaquet et al, 1996). However, when data from two patients who received the highest dose, and had the highest number of concentration measurements, were analysed individually it was not possible to characterize the parameters of a triexponential model. Moreover, the initial distribution half-life of 8 min was similar to previous reports in the range of 3–5 min (Jaquet et al, 1996). Inclusion of dose as a factor affecting apparent V1 produced population predicted concentrations that were closer to the measured concentrations suggesting that this problem might be dose-related. Figure 3 shows a trend towards higher bound to free doxorubicin ratios and much wider variability at higher doses. This suggests that the apparent dose effects on both bound and free doxorubicin may be related to variability in the formulation.

The only clinical factor found to influence free doxorubicin pharmacokinetics was height, which had a small effect on apparent V2. The elimination half-life of 85 h identified in this analysis was longer than the typical values of 20–50 h reported in many studies with doxorubicin (Bronchud et al, 1990; Piscitelli et al, 1993; Dobbs et al, 1995; Jaquet et al, 1996). It is possible that these differences are influenced by sampling strategy. In most of the previous studies samples were collected up to 48 h after the dose whereas in the present study samples were collected for up to 15 days. Thirty-two free doxorubicin measurements were available beyond 48 h post dose which might permit identification of a longer elimination half-life. Alternatively, the slow elimination may have been influenced by release of free doxorubicin from the PK1 complex or from cells.

Parameter estimates obtained from the free doxorubicin analysis were 1000 times higher than the bound estimates, reflecting the 1000-fold difference in concentrations and the very low availability of free doxorubicin in the formulation. The ratio of bound to free doxorubicin concentration at the time of the first sample was highly variable, especially at higher doses (Figure 3) and tended to increase then decrease during the dosage interval. This probably reflects the differences in pharmacokinetic profiles between the two species. Free doxorubicin had a very rapid distribution phase with a half-life of 8 min while the distribution of bound doxorubicin was slower with a median estimate of 2.7 h. In contrast, elimination half-lives were shorter for bound doxorubicin (49 h compared to 85 h). These observations, coupled to the detection of

© 1999 Cancer Research Campaign free doxorubicin at the first sample time (5–15 min after the start of the infusion) and the highly variable ratio, suggest that very small, but variable, amounts of free doxorubicin were present in the formulation at the time of administration.

One outlier was identified who had unexpectedly high free doxorubicin concentrations (population predicted peak 82 ng $ml^{-1}$, measured peak 441 ng $ml^{-1}$) and low bound concentrations (population predicted peak 104.3 µg $ml^{-1}$, measured peak 18.4 µg $ml^{-1}$). These unusual concentrations were associated with highest estimates of bound and lowest estimates of free parameters and probably contributed to the high intersubject variabilities in volume that were identified. The results for this patient suggest that some dissociation of the compound had occurred or that there was rapid cleavage of a small amount of PK1 at the time of administration. However, the latter explanation is unlikely because all the free doxorubicin profiles clearly followed a biexponential decline and there was no evidence of increasing concentrations after the infusion. The shape of the free doxorubicin profiles also suggested that free doxorubicin was not being leached from cells after intracellular cleavage. If that had been the case, a gradual increase in concentration might have been expected rather than simply a decline.

In summary, the pharmacokinetics of bound and free doxorubicin were determined from data collected during a phase 1 study of PK1. The population methodology allowed data from all patients, including those for whom only a few concentrations were available, to contribute to parameter estimation. Concentrations of bound and free doxorubicin differed by a factor of 1000 and this translated into population clearance estimates of 0.194 1 $h^{-1}$ for bound and 180 1 $h^{-1}$ for free doxorubicin. There was no evidence of dose dependency in clearance and although no clinical factors strongly influenced the clearances of bound or free doxorubicin, none of the patients had evidence of severe renal or, more importantly, hepatic impairment.

### REFERENCES

Amantea MA, Forrest A, Northfelt DW and Mamelok R (1997) Population pharmacokinetics and pharmacodynamics of pegylated-liposomal doxorubicin in patients with AIDS-related Kaposi's sarcoma. *Clin Pharmacol Ther* **61**: 301–311

Beal SL, Sheiner LB and Boeckmann AJ (1994) NONMEM IV User's Guide, Parts I–VI, NONMEM Project Group, University of California, San Francisco

Bronchud MH, Margison JM, Howell A, Lind M, Lucas SB and Wilkinson PM (1990) Comparative pharmacokinetics of escalating doses of doxorubicin in patients with metastatic breast cancer. *Cancer Chemother Pharmacol* **25**: 435–439

Bruno R, Vivier N, Vergniol JC, DePhillips SL, Montay G and Sheiner LB (1996) A population pharmacokinetic model for taxotere: model building and validation. *J Pharmacokinet Biopharm* **24**: 153–172

Chabot GG, Abigerges D, Catimel G, Culine S, Deforni R, Extra JM, Mahjoubi H, Herait P, Armand JP, Bugat R, Clavel M and Marty ME (1995) Population pharmacokinetics and pharmacodynamics of irinotecan (CPT-11) and active metabolite SN-38 during Phase I trials. *Ann Oncol* **6**: 141–151

Chatelut E, Canal P, Brunner V, Chevreau C, Pujol A, Boneu A, Roche H, Houin G and Bugat R (1995) Prediction of carboplatin clearance from standard morphological and biological patient characteristics. *J Natl Cancer Inst* **87**: 573–580

Cockcroft DW and Gault MH (1976) Prediction of creatinine clearance from serum creatinine. *Nephron* **16**: 31–41

Dobbs NA, Twelves CJ, Gillies H, James CA, Harper PG and Rubens RD (1995) Gender affects doxorubicin pharmacokinetics in patients with normal liver biochemistry. *Cancer Chemother Pharmacol* **36**: 473–476

Duncan R, Cable HC, Lloyd JH, Rejmanova P and Kopecek J (1982) Degradation of side chains of N-(2-hydroxypropyl)methacrylamide) copolymers by lysosomal thioproteinases. *Bioscience Rep.* **2**: 1041–1046

Fraier D, Frigerio E, Pianezzola E, Strolin Benedetti M, Cassidy J and Vasey P (1995) A sensitive procedure for the quantitation of free and N-(2-hydroxypropyl) methacrylamide polymer-bound doxorubicin (PK1) and some of its metabolites, 13-dihydrodoxorubicin, 13-dihydrodoxorubicine and doxorubicinone, in human plasma and urine by reversed-phase HPLC with fluorimetric detection. *J Pharm Biomed Anal* **13**: 625–633

Jacquet J-M, Bressolle F, Galtier M, Bourrier M, Donadio D, Jourdan J and Rossi J-F (1996) Doxorubicin and doxorubicinol: intra- and inter-individual variations of pharmacokinetic parameters. *Cancer Chemother Pharmacol* **27**: 219–225

Jodrell DI, Reyno LM, Sridhara R, Eisenberger MA, Tkaczuk KH, Zuhowski EG, Sinibaldi VJ, Novak MJ and Egorin MJ (1994) Suramin: development of a population pharmacokinetic model and its use with intermittent short infusions to control plasma drug concentration in patients with prostate cancer. *J Clin Oncol* **12**: 166–175

Launay Iliadis MC, Bruno R, Cosson V, Vergniol JC, Oulidaissa D, Marty M, Clavel M, Aapro M, Lebail N and Iliadis A (1995) Population pharmacokinetics of docetaxel during Phase I studies using nonlinear mixed effect modelling and nonparametric maximum likelihood estimation. *Cancer Chemother Pharmacol* **37**: 47–54

Maeda H and Matsumura Y (1989) Tumoritropic and lymphotropic principles of macromolecular drugs. *CRC Crit Rev Ther Drug Carrier Sys* **6**: 193–210

Matsumura Y and Maeda H (1986) A new concept for macromolecular therapeutics in cancer chemotherapy; mechanism of tumoritropic accumulation of proteins and the antitumor agent SMANCS. *Cancer Res* **46**: 6387–6392

McLeod HL, Graham MA, Aamdal S, Setanoians A, Groot Y, Lund B and EORTC Early Clinical Trials Group (1996) Phase I pharmacokinetics and limited sampling strategies for the bioreductive alkylating drug EO9. *Eur J Cancer* **32A**: 1518–1522

Nguyen L, Chatelut E, Chevreau C, Tranchand B, Lochon I, Bachaud JM, Pujol A, Houin G, Bugat R and Canal P (1998) Population pharmacokinetics of total and unbound etoposide. *Cancer Chemother Pharmacol* **41**: 125–132

Piscitelli SC, Rodvold KA, Rushing DA and Tewksbury DA (1993) Pharmacokinetics and pharmacodynamics of doxorubicin in patients with small cell lung cancer. *Clin Pharmacol Ther* **53**: 555–561

Reyno LM, Egorin MJ, Eisenberger MA, Sinibaldi VJ, Zuhowski EG and Sridhara R (1995) *J Clin Oncol* **13**: 2187–2195

Samara E and Granneman R (1997) Role of population pharmacokinetics in drug development. A pharmaceutical industry perspective. *Clin Pharmacokinet* **32**: 294–312

Schoemaker RC and Cohen AF (1996) Estimating impossible curves using NONMEM. *Br J Clin Pharmacol* **42**: 283–289

Tett SE, Holford NHG and McLachlan AJ (1998) Population pharmacokinetics and pharmacodynamics: an underutilised resource. *Drug Inf J* **32**: 693–710

Sheiner LB, Rosenberg B and Marathe VV (1977) Estimation of population characteristics of pharmacokinetic parameters from routine clinical data. *J Pharmacokinet Biopharm* **5**: 445–479

Yuh L, Beal S, Davidian M, Harrison F, Hester A, Kowalski K, Vonesh E and Wolfinger R (1994) Population pharmacokinetic/pharmacodynamic methodology and applications: a bibliography. *Biometrics* **50**: 566–575

Vasey PA, Kaye SB, Morrison R, Twelves C, Wilson P, Duncan R, Thomson AH, Murray L, Hilditch TE, Murray T, Burtles S, Fraier D, Frigero E and Cassidy J (1999) Phase I clinical and pharmacokinetic study of PK1 (HPMA CO-Polymer doxorubicin); first member of a new class of chemotherapeutic agents – drug-polymer conjugates. *Clin Cancer Res* **5**: 83–94

*© 1999 Cancer Research Campaign*
